# Supplementary figures and images for: Biomechanical study on the effect of atherosclerosis on the vulnerability of thoracic aorta, and it’s role in the development of traumatic aorta injury
Source: PLoS One. 2023 Sep 8;18(9):e0287652. doi: 10.1371/journal.pone.0287652 (PMC10491303; doi:10.1371/journal.pone.0287652)

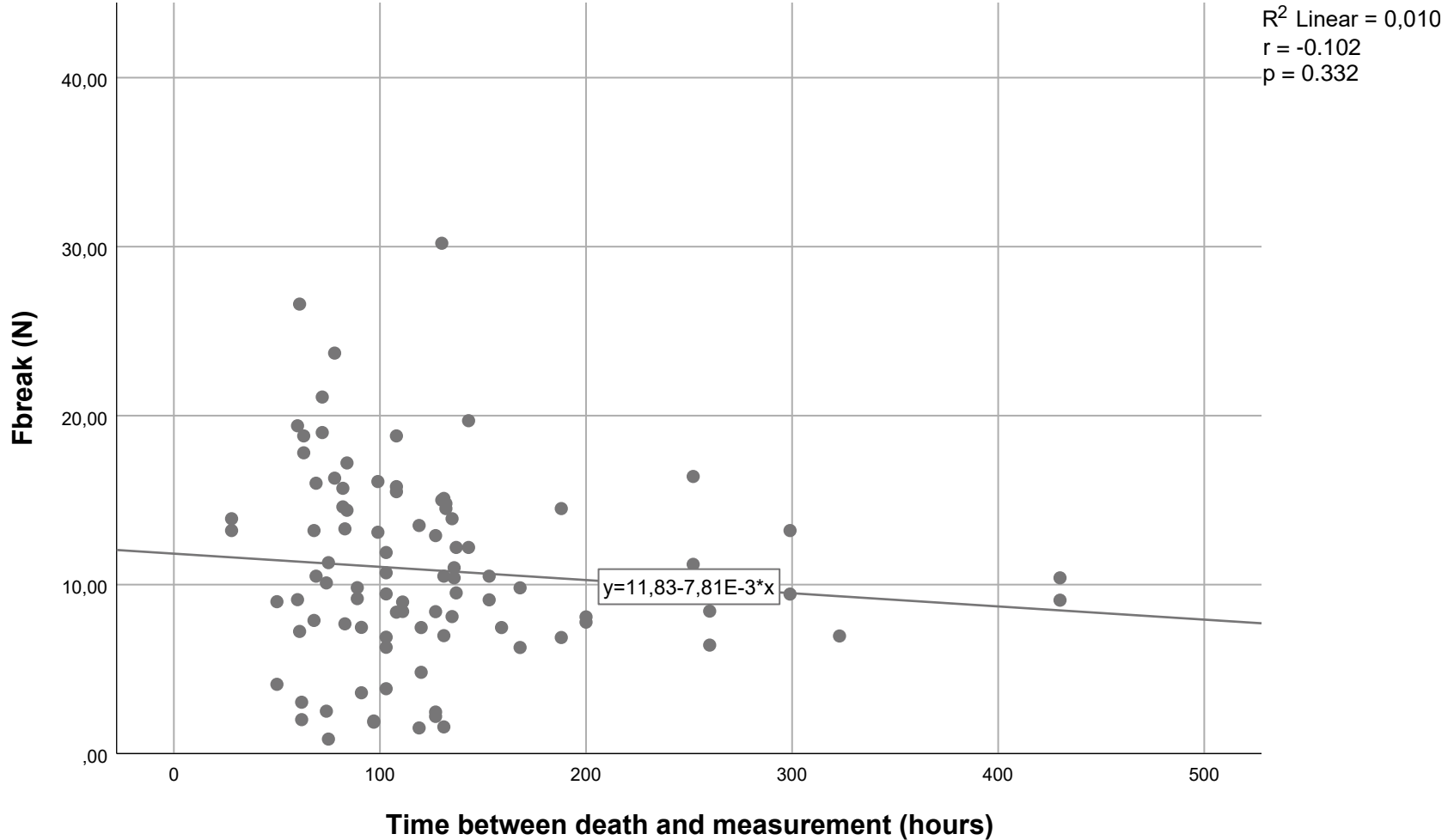

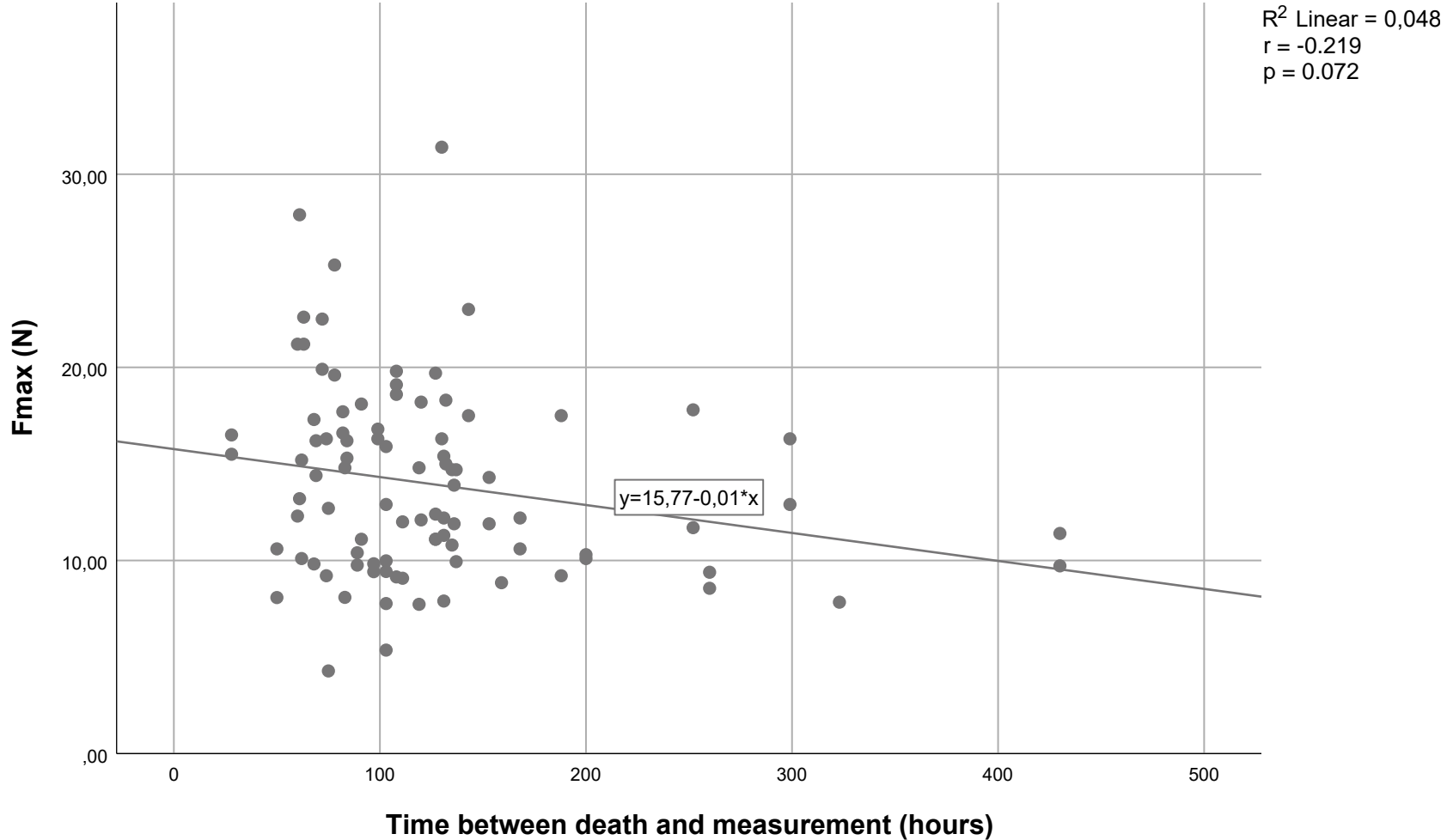

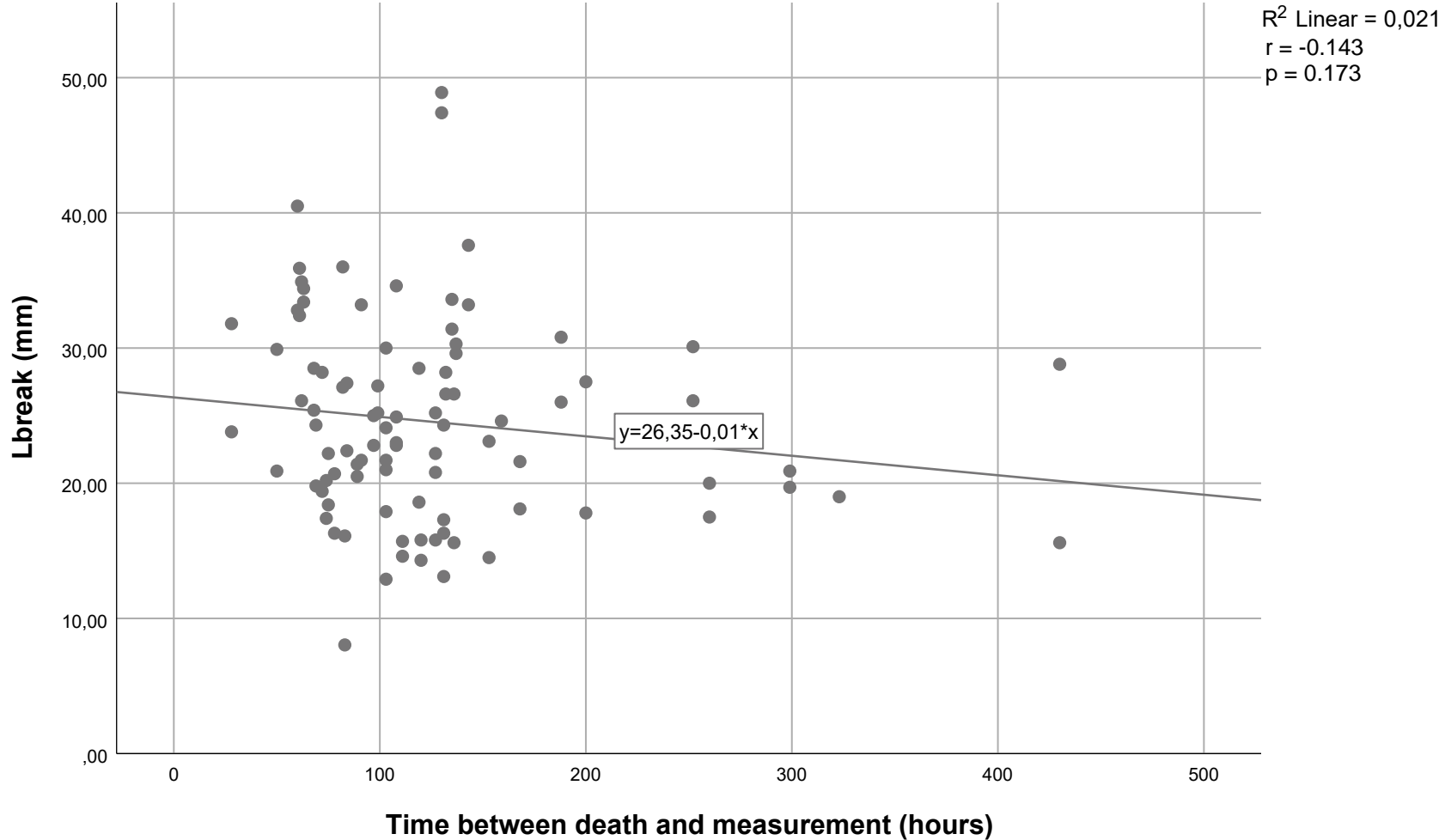

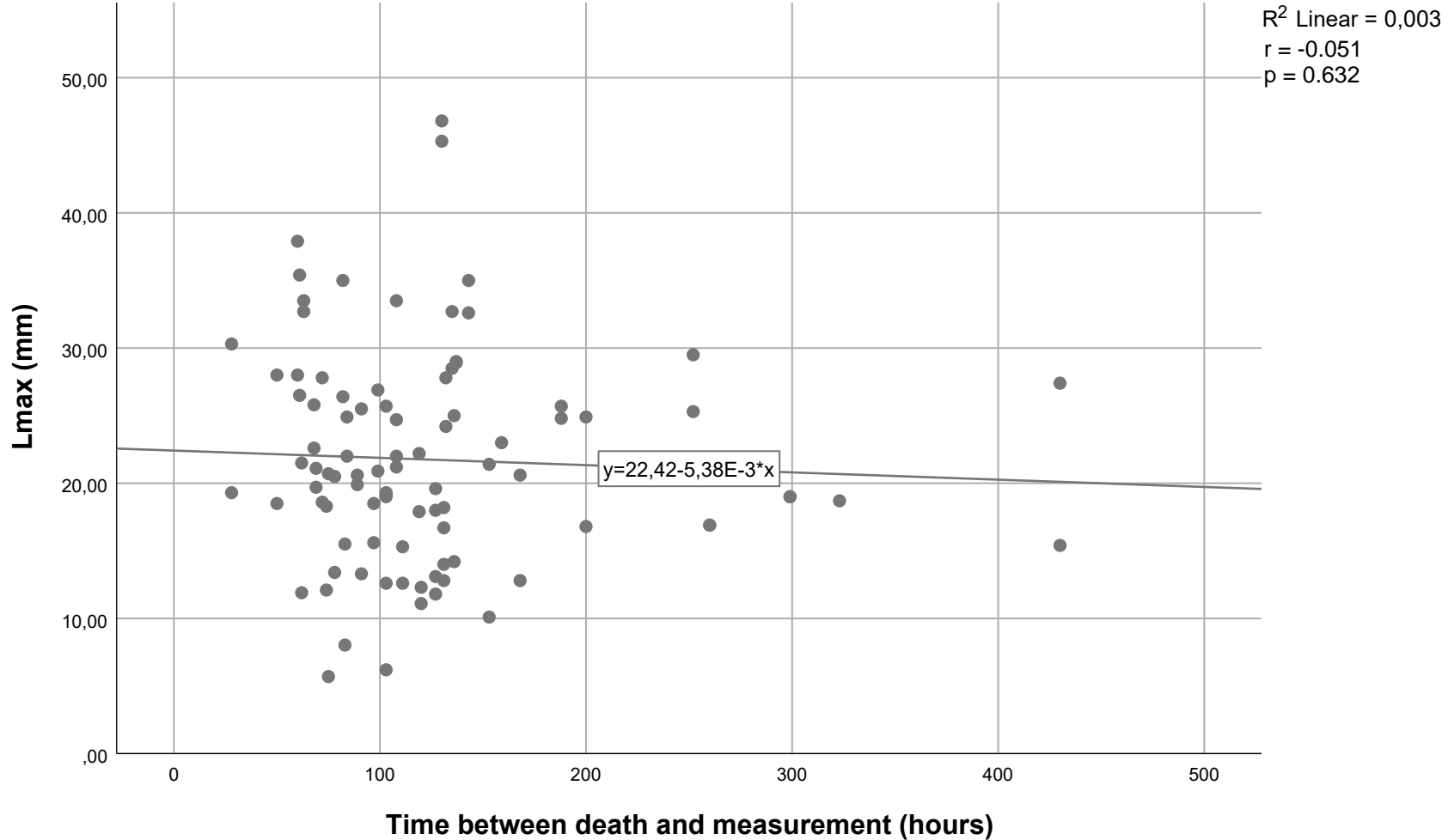

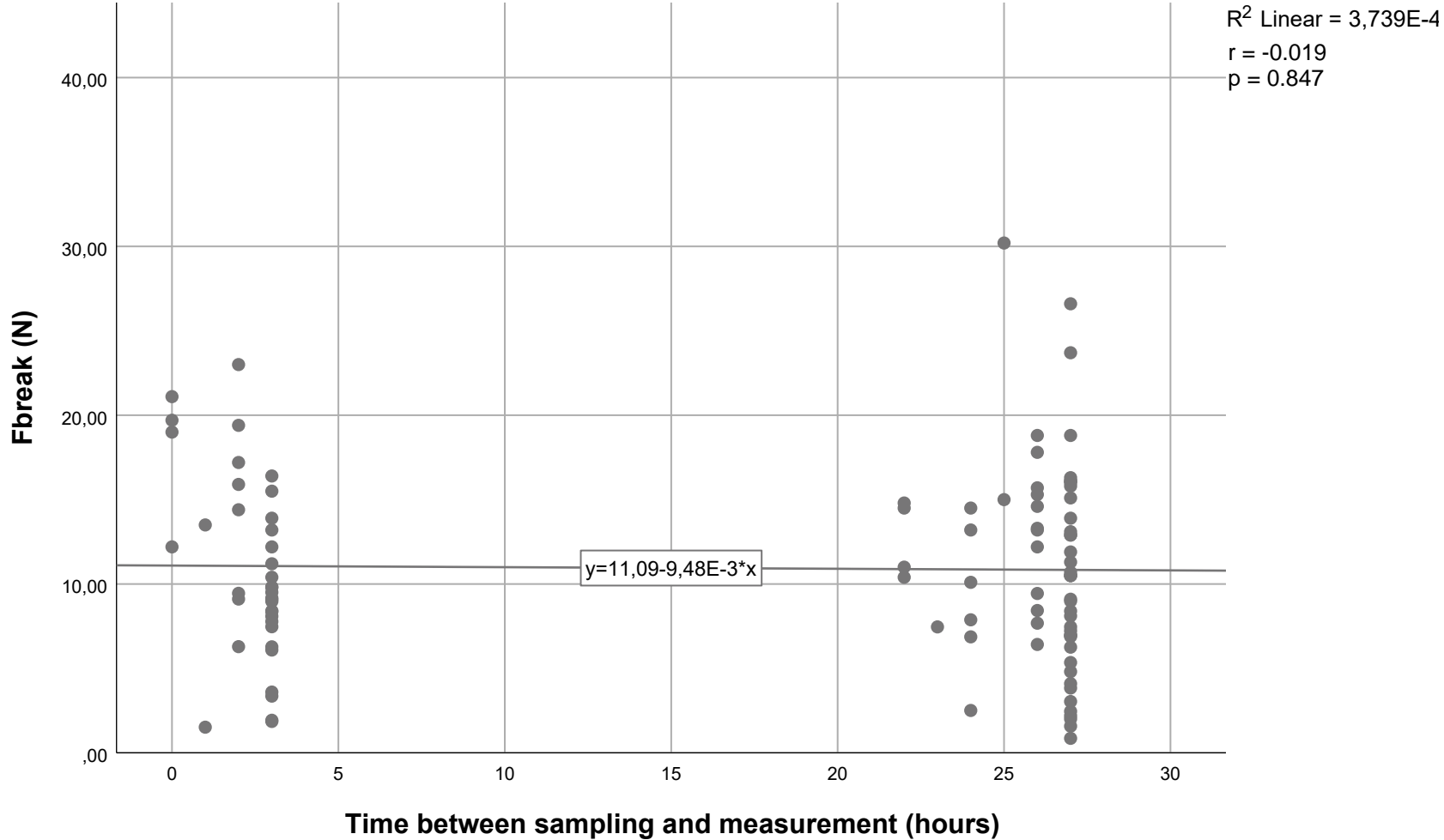

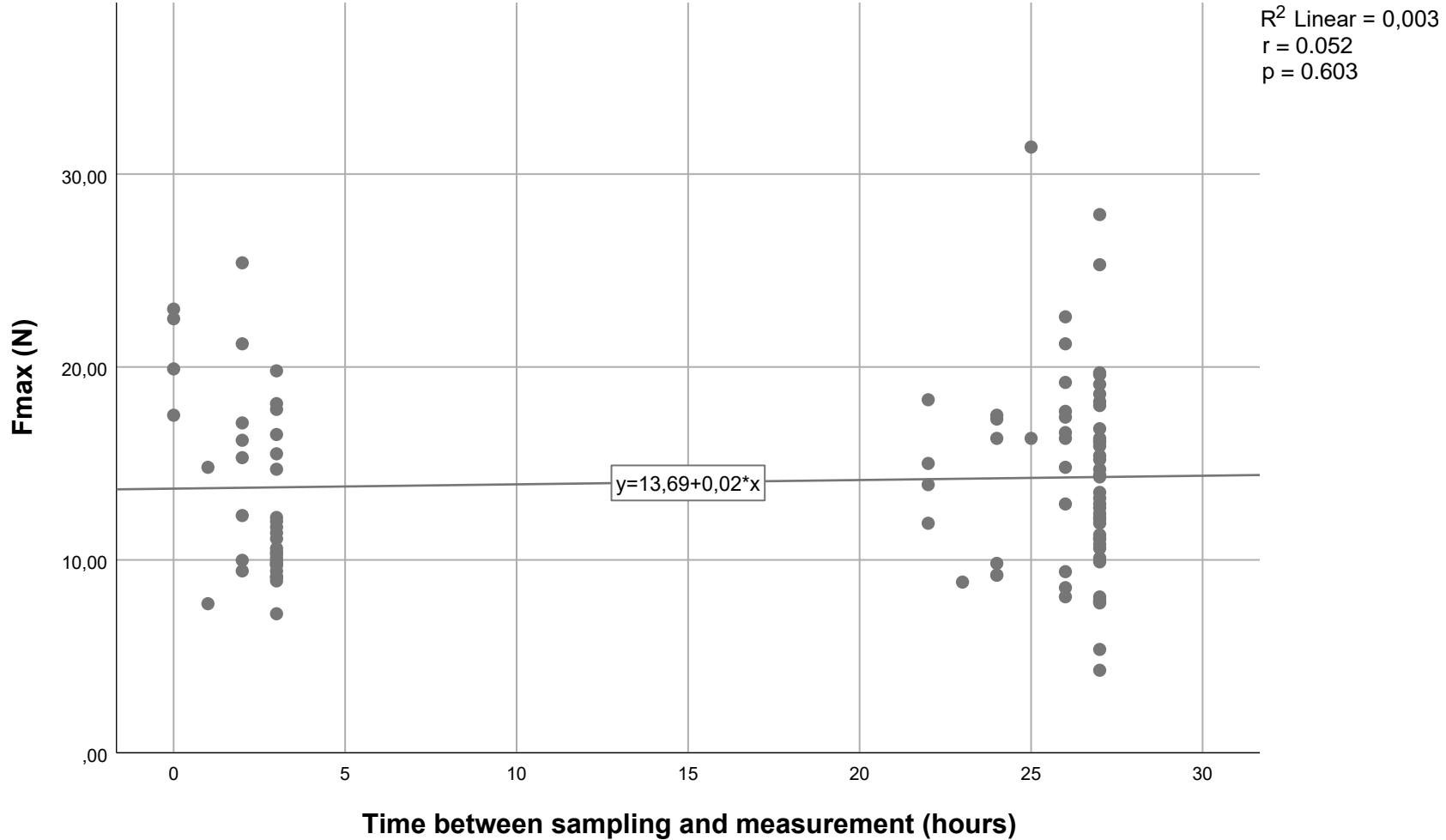

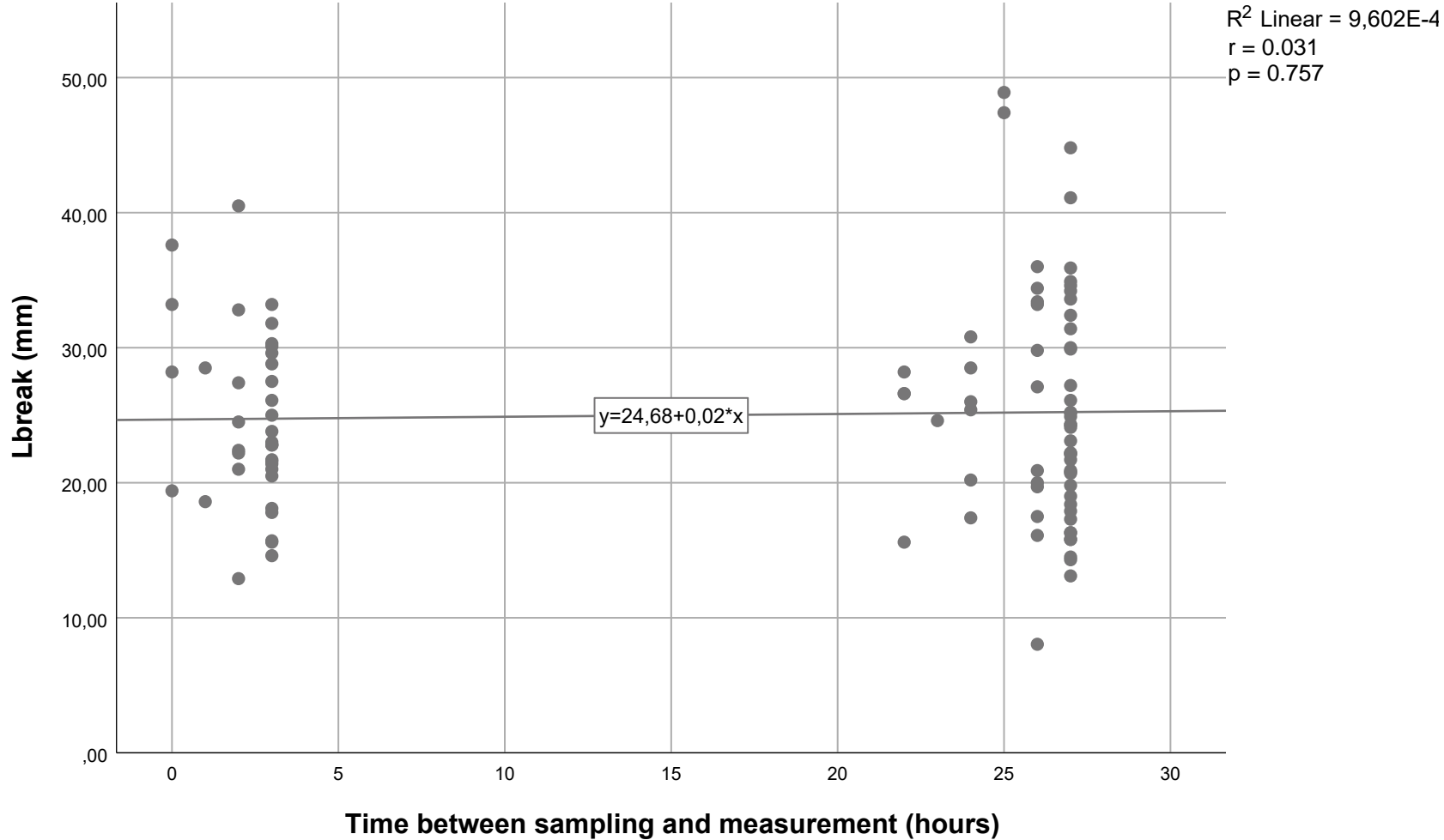

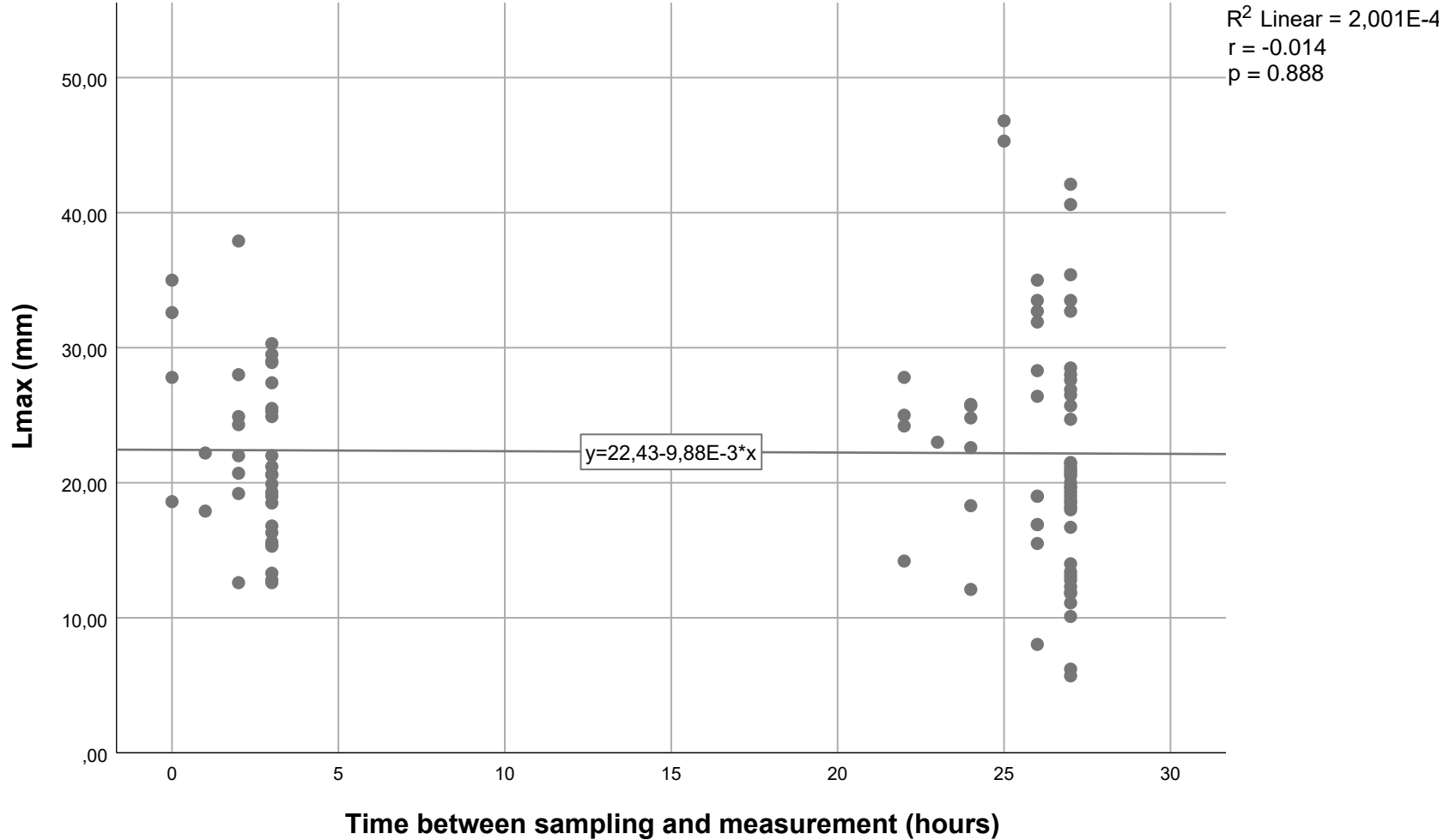

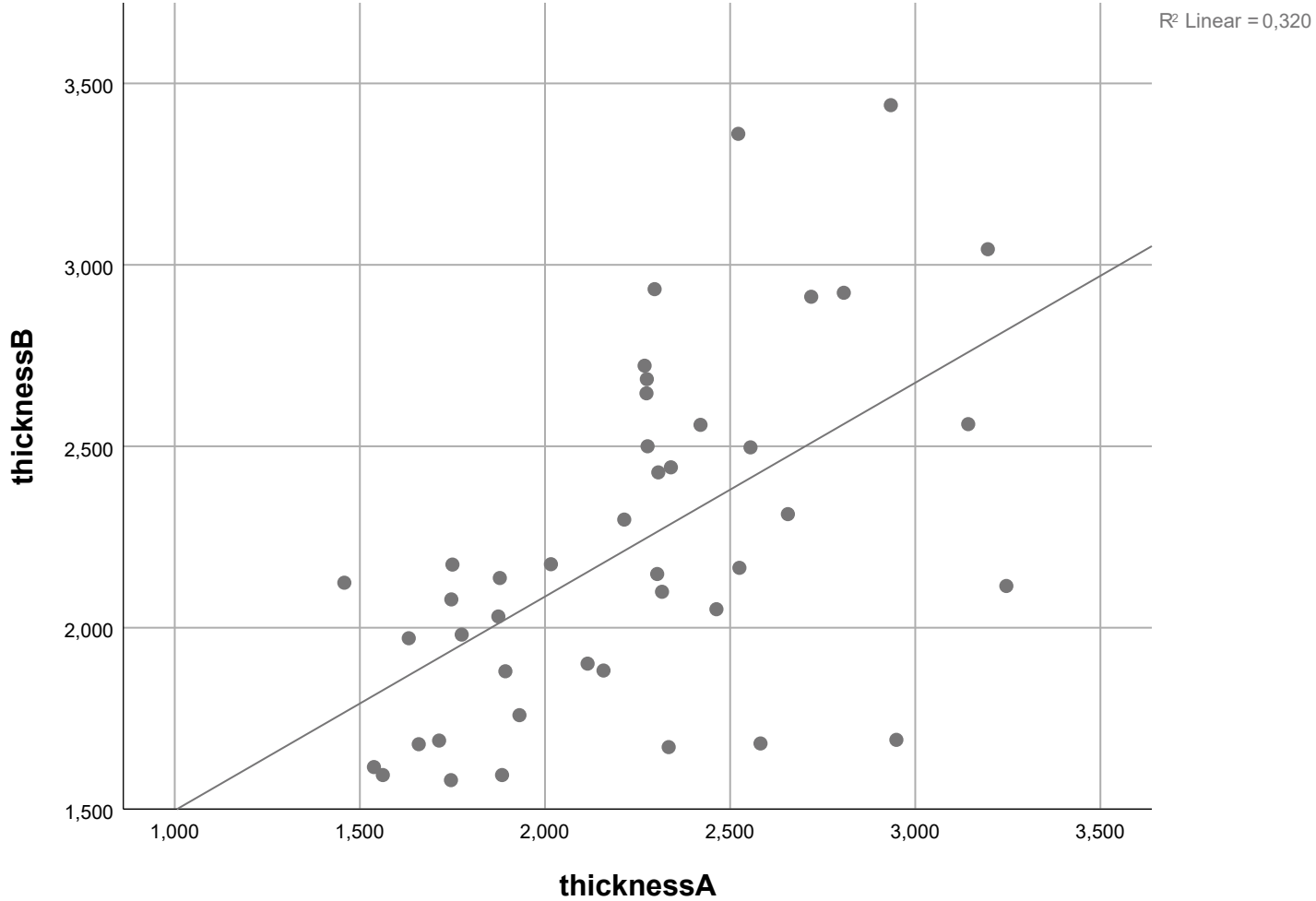

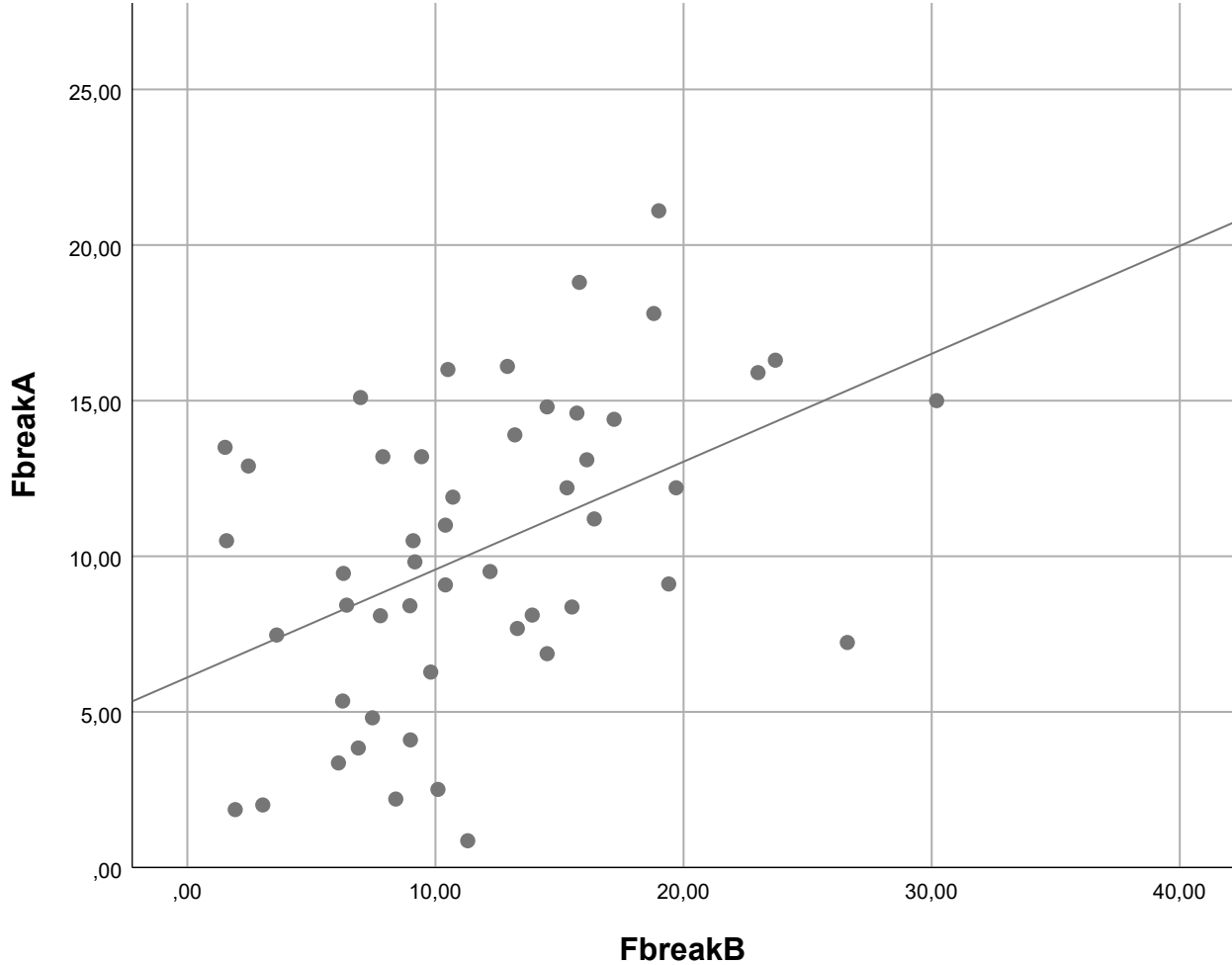

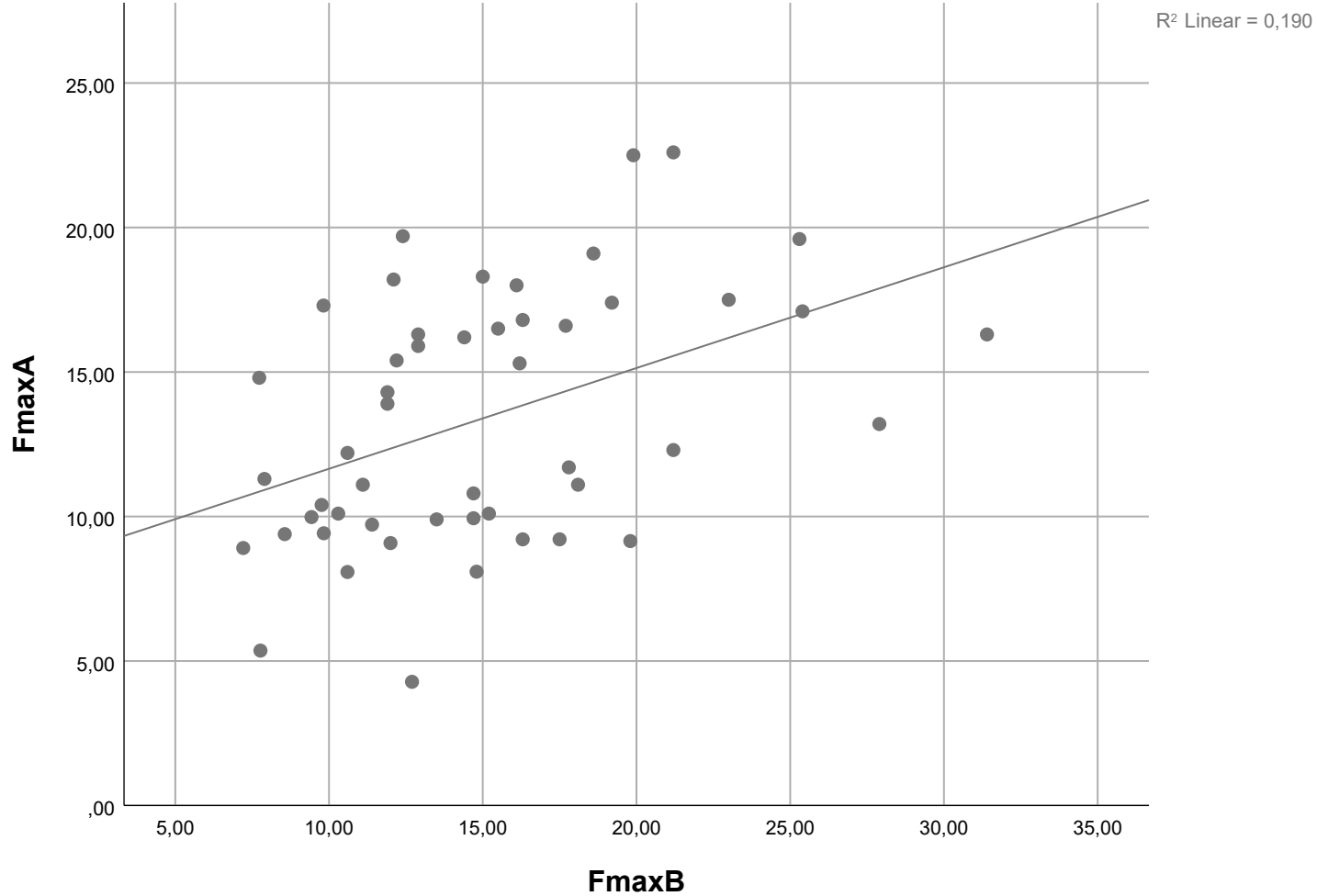

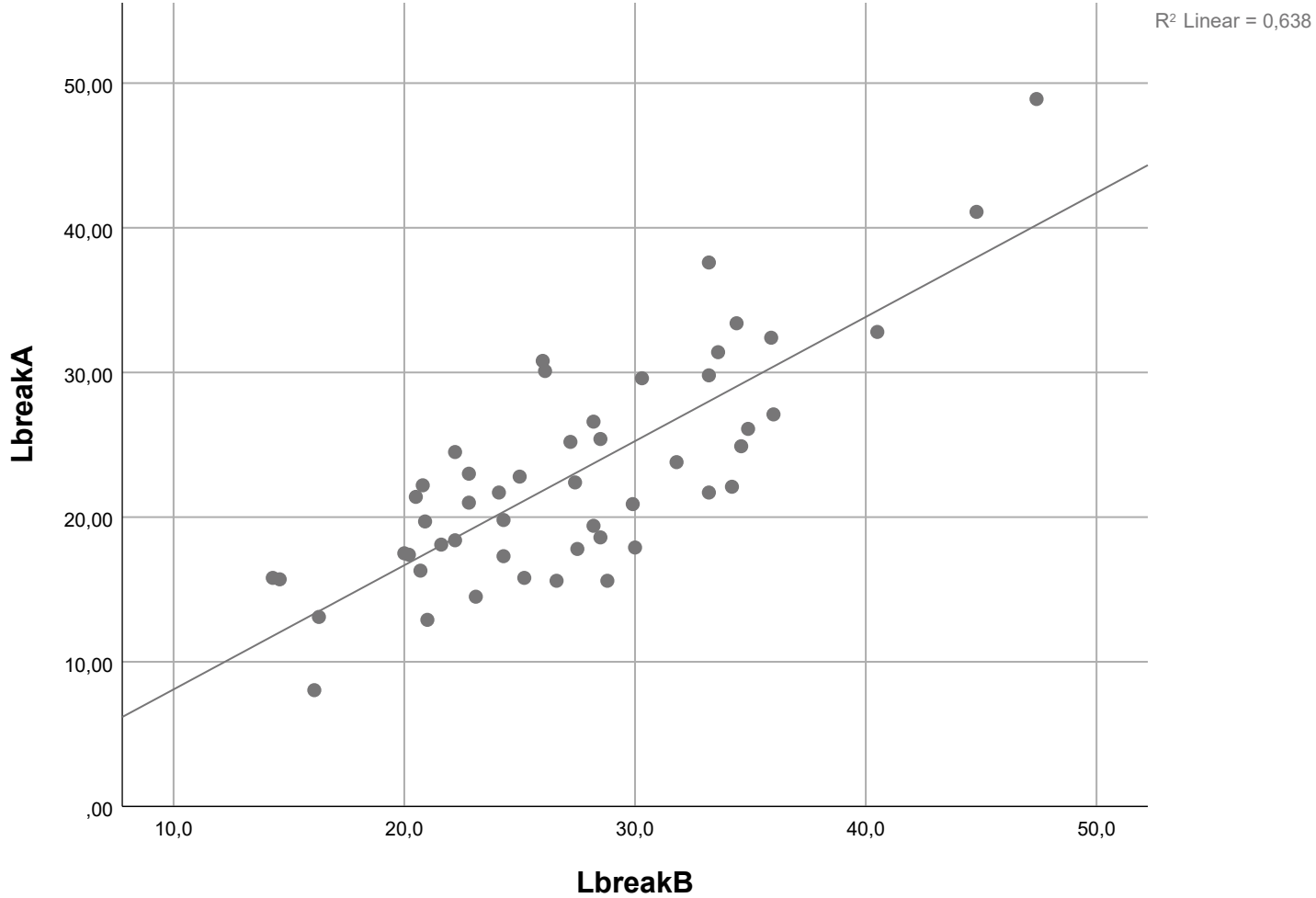

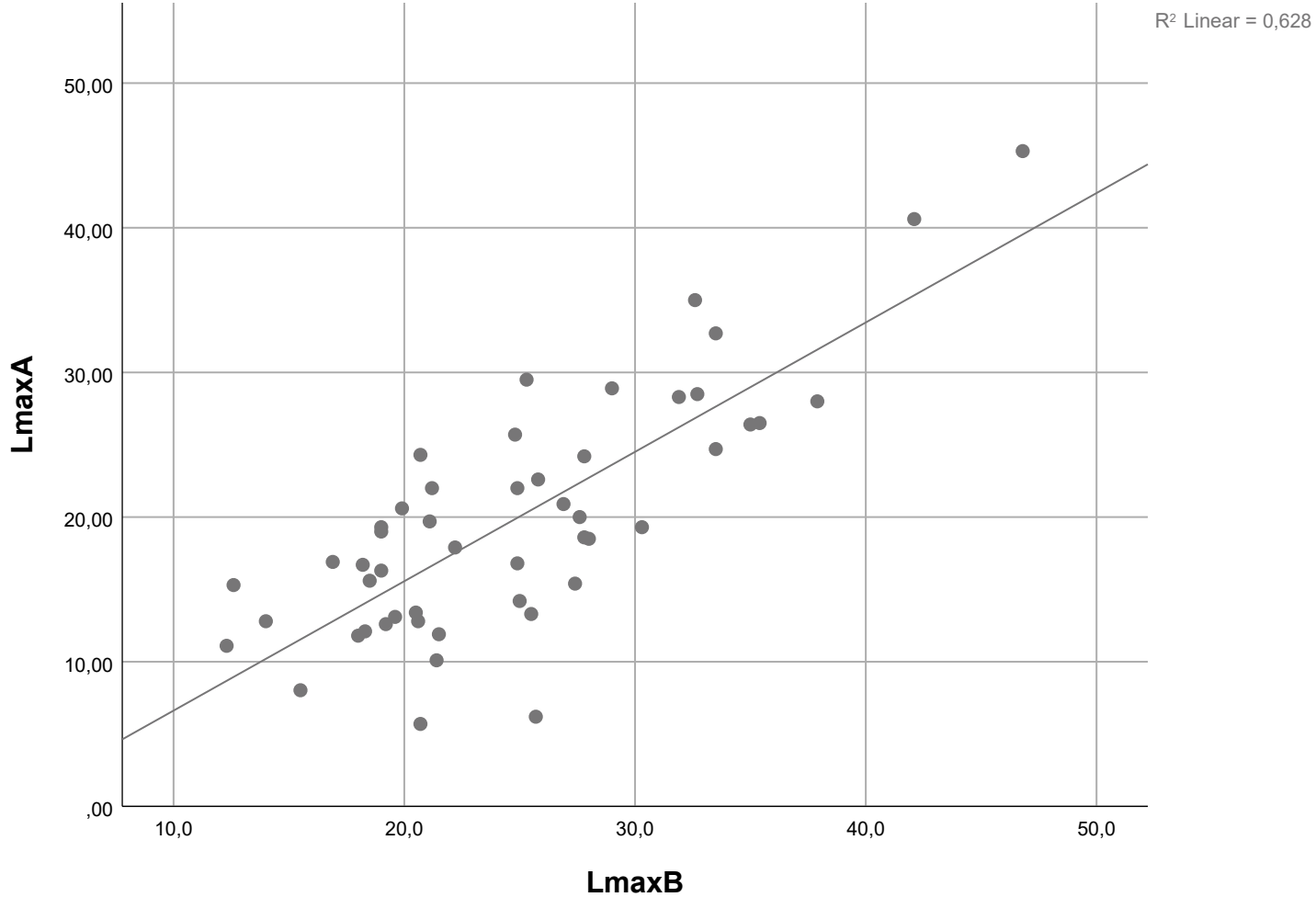

Supplement: S1 File — Electronic supplementary file containing the scatter plots, correlation coefficients and R^2 values of time between death and measurement, and the time between sample removal and measurement (Fmax, Lmax, Fbreak, Lbreak). No correlation was found between the parameters. (PDF) [file pone.0287652.s001.pdf]
