# Supplementary material for: Biomechanical study on the effect of atherosclerosis on the vulnerability of thoracic aorta, and it’s role in the development of traumatic aorta injury
Source: PLoS One. 2023 Sep 8;18(9):e0287652. doi: 10.1371/journal.pone.0287652 (PMC10491303; doi:10.1371/journal.pone.0287652)
Supplement: Attachment — Submitted filename: Answer to Reviewers 2.docx [file pone.0287652.s004.docx]

**Answer to Reviewers**

On behalf of all the authors, I would like to thank all of you for reviewing our
manuscript entitled “***Biomechanical study on the effect of atherosclerosis on the vulnerability of thoracic aorta, and it’s role in the development of traumatic aorta injury***”. The remarks were valid, and greatly helped to correct the manuscript. Based on your profound reviews we think we was able to greatly increase the quality of our manuscript. We hope you will find our corrected manuscript worth publishing.

We uploaded a “track changes” version of the manuscript also (following the PlosOne guideline for revisions).

Below you can find our detailed answers to your remarks and questions. The missing text reference to Figure 3 (Fig 3) not previously noticed was also corrected.

**Comment to Reviewer 1.**

Thank you for your support of our manuscript, and thank you for the review, since your contribution as a reviewer greatly enhanced the quality of our manuscript.

**Comment and answer to Reviewer 1.**

Thank you for your support of our manuscript. We have found all your comments and suggestions well founded and helpful. Addressing the problems you highlighted and making the structural change you suggested was necessary for making a manuscript with a higher standard. Here you can found the answers to your further comments/suggestion.

*The resolution of several of the graphics are still prohibitively poor, at least when printed. Regarding question 16, transposing and 'transporting' a table is not the same, but this may not be in the authors interest to do.*

Graphics were created for online viewing (as Plos One is an online journal). Printing will lead to the shrinkage in size which may decrease the quality (but we think the main reason of the decrease in quality is due to how the submission system generate the pdf files – you can compare it with the original picture we gave you the links). The Figure quality was checked and approved by Plos One’s PACE tool. The table was relocated of course, not transposed.

*In the discussion the authors write that "the rupture occurs at 2/3 of force if the aorta" is calcified" (not verbatim), and attribute this effect to "the weakening structure of the intima". I have always been under the impression that the intima is not so load-bearing in blood vessels. I think the authors could expand their reasoning here. Otherwise I do not have more comments.*

The intima is not the main load-bearing part of blood vessels, but still it has an important function also in this regard. It is proven by the previous observation by different authors (citation 16, 17, 18) that the aortic rupture due to blunt force always starts in the intima. We can hypothesize, that the rupture of the intima decrease the integrity of the vessel wall at that point, where the rupture can continue (spread) easily to the other layers. We expanded the reasoning in the discussion section.

Thank you again your reviews.

Yours sincerely:

Authors
